# Phase determination using chromosomal microarray and fluorescence in situ hybridization in a patient with early onset Parkinson disease and two deletions in *PRKN*

**DOI:** 10.1002/mgg3.386

**Published:** 2018-03-25

**Authors:** Eli S. Williams, Matthew J. Barrett, Radhika Dhamija, Lisa Toran, Chelsea Chambers, Mani S. Mahadevan, Wendy L. Golden

**Affiliations:** ^1^ Department of Pathology University of Virginia Charlottesville VA USA; ^2^ Department of Neurology University of Virginia Charlottesville VA USA; ^3^ Departments of Clinical Genomics and Neurology Mayo Clinic Phoenix AZ USA

**Keywords:** cytogenetics, DNA copy number variations, early‐onset, genetic testing, microarray analysis, parkin, Parkinson disease, PRKN

## Abstract

**Background:**

Mutations in the parkin gene (*PRKN*) are the most commonly identified genetic factors in early onset Parkinson disease (EOPD), with biallelic mutations, resulting in a clinical phenotype. However, normal variation is also common in *PRKN*, particularly in the form of copy number variation (CNV), challenging interpretation of genetic testing results. Here we report a case of a 29‐year‐old male with EOPD and two deletions in *PRKN* detected by chromosomal microarray (CMA).

**Methods:**

The proband was clinically examined by a neurologist for postural instability with frequent falls, bradykinesia, gait freezing with festination, and hypophonia. Chromosomal microarray analysis (CMA) was performed on the proband and his parents using the Affymetrix CytoScan HD microarray. Subsequent fluorescence in situ hybridization (FISH) was performed on the proband and both parents.

**Results:**

Chromosomal microarray detected the presence of two deletions of *PRKN* in the proband. Parental CMA analysis was performed to determine the clinical significance of this finding, as well as to demonstrate phase of these deletions. Parental CMA revealed that one deletion was paternally inherited and one deletion was de novo. A custom FISH approach was then successfully used to phase the deletions.

**Conclusion:**

Chromosomal microarray and fluorescence in situ hybridization analysis of this trio identified two deletions in *PRKN* occurring in trans, providing a genetic etiology for the clinical diagnosis of EOPD. The determination of inheritance and phase of the deletions was critical to the proper interpretation of these results. These findings highlight the utility of CMA in the detection of clinically relevant CNVs in cases of EOPD, and also serve to emphasize the importance of follow‐up FISH and parental testing.

## 1 INTRODUCTION

Parkinson disease is a common neurodegenerative disease, affecting approximately 1% of individuals over the age of 65 (de Rijk et al., 2000). The disease is characterized by the cardinal features of bradykinesia, resting tremor, and muscular rigidity. Although Parkinson disease is age‐related and is more prevalent in older populations, approximately 10% of Parkinson disease is diagnosed before the age of 50 (Mehanna, Moore, Hou, Sarwar, & Lai, 2014). These cases are known as early onset Parkinson disease (EOPD) and are more often attributable to genetic abnormalities than the classic form of Parkinson disease. The parkin gene (*PRKN*, formerly *PARK2*, OMIM# 602544) (Kitada et al., 1998) is the most commonly mutated gene in EOPD, accounting for approximately half of all mutations detected in individuals with EOPD (Abbas et al., 1999; Hedrich et al., 2004).

*PRKN* encodes the parkin protein, an E3 ubiquitin ligase, important for the degradation of damaged mitochondria among other roles. It is believed that *PRKN* variants impair mitochondrial homeostasis, ultimately leading to neuronal cell death (Scott, Dawson, & Dawson, 2017). Biallelic mutations in *PRKN* are required for a clinical phenotype (Abbas et al., 1999; Kitada et al., 1998). The mutation spectrum of *PRKN* is broad, with point mutations and copy number variation (ie deletions and duplications) found at a similar frequency (Abbas et al., 1999; Hedrich et al., 2004). Due to the relatively high frequency of copy number variation (CNV) within *PRKN*, chromosomal microarray (CMA) is a robust approach for the detection of clinically‐relevant genetic variants resulting in EOPD. However, CMA studies of normal individuals have revealed numerous CNV polymorphisms within *PRKN* (MacDonald, Ziman, Yuen, Feuk, & Scherer, 2014). Moreover, CMA is unable to determine whether CNVs occur on the same allele (cis) or on opposite alleles (trans) necessitating follow up studies.

Herein, we report a 29‐year‐old male with EOPD and two *PRKN* deletions identified by CMA. Follow‐up fluorescence in situ hybridization (FISH) studies of the proband and parents demonstrated that these deletions occurred in trans, providing a genetic etiology for the clinical diagnosis of EOPD.

## 2 CLINICAL REPORT

A 29‐year‐old male presented with postural instability with frequent falls, bradykinesia, gait freezing with festination, and hypophonia. Beginning at age 20, he developed postural instability, gait imbalance and freezing of gait. Over time, his postural instability led to weekly falls. He had progressive bradykinesia leading to an inability to feed himself, and he exhibited small amplitude bilateral hand tremors. The patient reported that his voice became progressively softer and higher pitched beginning around age 25.

At the time of his first evaluation at age 27, he was noted to have severe parkinsonism with masked facies, high‐pitched, hypophonic and slowed speech, mild bilateral resting tremor, rigidity in the bilateral upper extremities, and bradykinesia with finger tapping and other rapid successive movements. Bradykinesia was characterized by decrement over time and arrests in movement. He also had diffuse hyperreflexia. His gait was wide‐based, unsteady, slow and notable for dystonic posturing in the right foot. He had severe postural instability and severe difficulty with activities of daily living. An MRI of the brain was normal. Ceruloplasmin, vitamin B12, thyroid function, electrolytes, renal function, hepatic function, complete blood count, and GAD65 antibody were all normal. Family history was negative for similarly affected individuals.

He was started on carbidopa/levodopa and experienced dramatic improvement in all motor symptoms, and he was eventually able to obtain employment. Treatment was complicated by levodopa‐induced dyskinesias, even at low doses of carbidopa/levodopa, and he had persistent right leg dystonia.

As he reportedly had poor performance in school, chromosomal microarray analysis (CMA) was ordered as an initial genetic evaluation.

## 3 METHODS

Ethical Compliance: This study was performed under waiver of approval HSR# 20492 from the University of Virginia IRB for Health Sciences Research.

DNA was extracted from peripheral blood specimens, using the MagNAPure Compact Instrument for nucleic acid purification following the manufacturer's protocol (Roche Life Science, Indianapolis, IN, USA). CMA was performed, using the Affymetrix CytoScan HD Array following the manufacturer's protocol (Affymetrix, Santa Clara, CA, USA). Copy number variations larger than 50 kilobases and containing at least 25 markers were evaluated for clinical significance. All genomic coordinates are given according to Hg19, *PRKN* reference sequence: NM_004562.2.

Fluorescence in situ hybridization analysis was performed on metaphase chromosomes, using standard techniques. The BAC probe used for the larger deletion was RP11‐307K1 (chr6:162,945,961‐163,144,665) and was labeled with fluorescein (green). A custom fosmid probe was designed for the smaller deletion, W12 1336G3 (chr6:162,668,984‐162,706,732) which was labeled in ROX (red). Localization studies for each probe were performed, using a 6p subtelomere probe as a control. Metaphase FISH analysis demonstrated hybridization of each custom probe to the same chromosome as the control probe, indicating that both custom probes localize to chromosome 6, as expected (data not shown). Five metaphase cells were examined from the proband, his mother, and his father along with a normal control.

## 4 RESULTS

**Figure 1:**
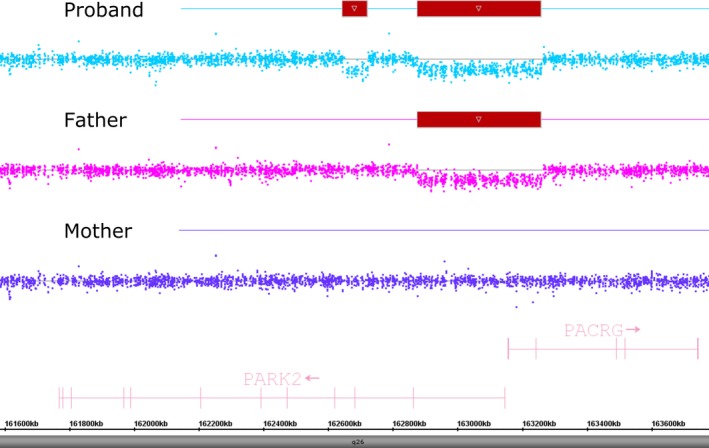
Chromosome microarray analysis of the proband and his parents. CMA of the proband revealed two deletions involving the PARK2 gene located at 6q26. The deletions are identified by a downshift of the probes in the deleted regions, further highlighted by the boxes above the involved probes. The paternal CMA demonstrates the presence of the larger deletion only, while the maternal CMA demonstrates normal copy number in this region

Chromosomal microarray of the proband detected two deletions involving *PRKN*. The first deletion is 381 kilobases (kb) in size and includes exon 1 of *PRKN*, as well as exons 1 and 2 of *PACRG* (chr6:162,878,218‐163,259,565). The second deletion is 71 kb in size and includes only exon 3 of *PRKN* (chr6:162,651,796‐162,722,581) (Figure 1).

To help determine the clinical significance of this finding, and to determine the phase of the deletions, parental CMA was performed. CMA analysis of the father revealed that he carried the larger deletion, including exon 1 of *PRKN* and exons 1 and 2 of *PACRG*. CMA analysis of the mother did not reveal any CNVs of *PRKN* (Figure 1). These results indicate that the 71 kb deletion detected in the proband is a de novo event. To definitively establish phase of the deletions, FISH analysis was performed on the proband, his mother, and his father, using probes specific to the two deleted regions.

**Figure 2:**
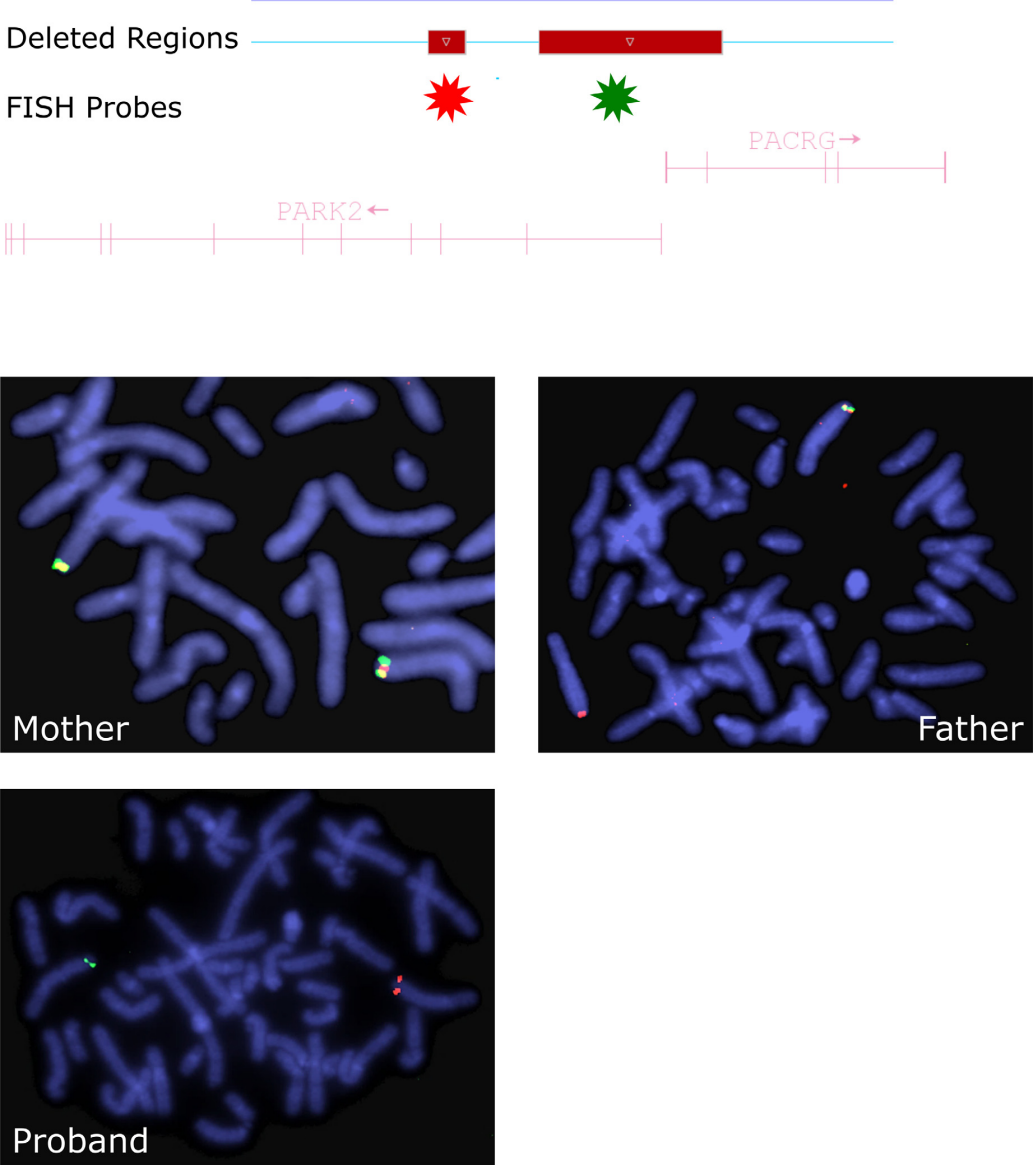
FISH analysis of the proband and his parents. Custom FISH probes were designed specific for the two deleted regions, with the probe for the smaller deletion labeled in red and the probe for the larger deletion labeled in green. Due to the close proximity of the deletions, a normal allele will demonstrate a yellow “fusion” signal, as demonstrated on in the maternal analysis. FISH analysis of the father demonstrated one allele with a yellow signal and one allele with a red signal, indicating a loss of the green probe. FISH analysis of the proband demonstrated one allele, using a red signal and one allele, using a green signal, indicating the presence of both deletions in trans

A BAC probe labeled in green (RP11‐307K1) was used to target the 381 kb deletion. A fosmid probe labeled in red (W12 1336G3) was used to target the 71 kb deletion. These two probes co‐localize on undeleted chromosomes, resulting in a yellow signal on a normal chromosome 6. Metaphase cells prepared from the mother (Figure 2) demonstrated a yellow signal on each chromosome 6, consistent with the normal control and her normal CMA result. Metaphase cells prepared from the father (Figure 2) demonstrated one yellow signal and one red signal, indicating one normal chromosome 6 and one chromosome 6 with a deletion including only the region including the RP11‐307K1 probe (green). Metaphase cells prepared from the proband demonstrated one red signal and one green signal, indicating that one chromosome 6 contained the 381 kb deletion and the homolog contained the 71 kb deletion. Therefore, FISH analysis successfully phased the deletions, demonstrating that they occurred in trans.

## 5 DISCUSSION

We report a case of EOPD in an individual with one inherited deletion and one de novo deletion occurring in trans in the *PRKN* gene. While compound heterozygous CNVs in *PRKN* have been previously described in EOPD (Kim et al., 2012), this case is illustrative for several reasons. First, this case demonstrates the utility of CMA in the detection of copy number variation contributing to Parkinson disease and Parkinsonian syndromes. Second, this case highlights the importance of appropriate follow up testing to the interpretation of CMA results in the context of autosomal recessive phenotypes.

Copy number variation comprises approximately half of the mutations detected in *PRKN* (Abbas et al., 1999; Hedrich et al., 2004), as well as a significant number of clinically‐relevant variants detected in other EOPD‐associated genes, such as *SNCA*,* PINK1*, and *DJ1* (Nuytemans, Theuns, Cruts, & Van Broeckhoven, 2010). Therefore, the ability of CMA to detect CNVs across the entire genome, including all relevant EOPD‐associated genes, makes it a powerful tool to screen for EOPD‐causing genetic abnormalities. CMA also allows clinicians to rule out common genetic etiologies for any additional features noted on patient examination. These features may include learning difficulties, such as those noted in our patient, speech or developmental delays, autism, and other indications that have been shown to be driven by CNV (Miller et al., 2010; Rehman, Dhamija, Williams, & Barrett, 2015).

While CMA serves as a useful first‐tier test for many clinical indications, several characteristics of CMA must be considered when interpreting results in the context of EOPD. Typically, CMA is unable to determine the phase of the CNVs, which is critical to the proper interpretation of results. The detection of two CNVs within *PRKN* or other autosomal recessive EOPD genes should lead to phase analysis to determine if both copies of the gene contain variants. In the clinical setting, heterozygous variation in *PRKN* has a variable interpretation ranging from a benign CNV to a variant of unknown significance. Several studies have associated heterozygous CNVs in *PRKN* with an increased susceptibility to EOPD, particularly in sporadic cases (Clark et al., 2006; Pankratz et al., 2009; Periquet et al., 2003). However, heterozygous copy number variation in *PRKN* is relatively common, and numerous CNVs are reported in unaffected individuals (MacDonald et al., 2014). The presence of the heterozygous *PRKN* deletion in the father of this case, an unaffected man of 62, reinforces the notion that heterozygous deletions of *PRKN* are unlikely to result in a clinical phenotype in the absence of other genetic abnormalities.

In this case, the presence of two deletions in the proband led to parental testing to help determine the clinical significance of the CMA results. Both parents were clinically unaffected at ages of 60 (mother) and 62 (father) at the time of testing. Parental CMA testing revealed the presence of the larger *PRKN* CNV in the father, while neither parent carried the smaller deletion. Therefore, phase analysis was incomplete following parental CMA testing. The sporadic nature of this case lends support to the finding of a de novo deletion in the proband. Subsequent FISH analysis was performed on this trio to definitively determine phase using custom designed FISH probes. This approach was able to clearly designate phase of the deletions as trans‐, demonstrating the strength of FISH analysis on metaphase chromosomes for CNV phase analysis. Biallelic deletions in PRKN are known to result in EOPD (Abbas et al., 1999; Kitada et al., 1998), and deletions encompassing only exon1 and only exon 3 have been previously described in affected individuals (Hedrich et al., 2004; Kim et al., 2012; Lucking et al., 2000). This case demonstrates that a standard clinical follow up including parental analysis via CMA and/or FISH offers a robust approach for determining the clinical significance of *PRKN* CNVs.

In summary, CMA plus appropriate follow‐up testing revealed the presence of two CNVs in *PRKN* occurring in trans in a 29 year old male with EOPD. Parental studies revealed the inheritance of one deletion from the father, while the second deletion occurred de novo. The determination of a de novo deletion in the proband substantially reduces the chance of another affected family member.

## CONFLICTS OF INTEREST

None declared.
